# Incidence and risk factors of hyperoxemia among potentially critically ill patients treated in hospital wards: a retrospective study

**DOI:** 10.55730/1300-0144.6048

**Published:** 2025-06-27

**Authors:** Mehmet Nuri YAKAR, Doğukan ŞENBERBER, Ozan BALKABAK, Nurgazy SHERMATOV, Kaan KÖŞKER, Selin ELDEN, Emel İBİŞOĞLU, Begüm ERGAN, Volkan HANCI, Necati GÖKMEN

**Affiliations:** 1 Division of Intensive Care, Department of Anesthesiology and Reanimation, Şişli Hamidiye Etfal Training and Research Hospital, University of Health Sciences, İstanbul, Turkiye; 2 Department of Anesthesiology and Reanimation, Faculty of Medicine, Dokuz Eylül University, İzmir, Turkiye; 3 Department of Chest Diseases, Faculty of Medicine, Dokuz Eylül University, İzmir, Turkiye

**Keywords:** Critical illness, critical care, hyperoxemia, intensive care units, oxygen, respiratory therapy

## Abstract

**Background/aim:**

The literature shows a link between hyperoxemia and poor outcomes, whereas this association remains unclear in hospital wards. This study aims to determine the incidence of hyperoxemia in hospital wards and its risk factors.

**Materials and methods:**

Patients aged ≥ 18 years who underwent an evaluation by an intensivist between 1 January 2020 and 31 December 2020, while receiving treatment in hospital wards, were included in the study following ethics committee approval. Patients with hypoxemia (partial pressure of oxygen [PaO_2_] < 60 mmHg), a condition related to Coronavirus disease 2019, a hospital stay < 1 day, or missing data were excluded. Patients were divided into two groups: normoxemia (60 mmHg ≤ PaO_2_ < 120 mmHg) and hyperoxemia.

**Results:**

The incidence of hyperoxemia was 42.2%. Patients with hyperoxemia had longer hospital stays and higher intensive care unit admission rates than those with normoxemia. Partial pressure of carbon dioxide < 30 mmHg (OR, 1.61; 95% CI, 1.16–2.25; p = 0.005), hemoglobin ≤ 10.3 g/dL (OR, 1.33; 95% CI, 1.01–1.75; p = 0.044), positive pressure ventilation (OR, 1.73; 95% CI, 1.09–2.74; p = 0.021), fraction of inspired oxygen ≥ 50% (OR, 1.71; 95% CI, 1.10–2.65; p = 0.018), type IV respiratory failure (OR, 1.62; 95% CI, 1.05–2.51; p = 0.030), and receiving treatment on surgical units (OR, 1.47; 95% CI, 1.02–2.12; p = 0.038) were independently associated with hyperoxemia. Charlson comorbidity index > 6 (OR, 0.64; 95% CI, 0.49–0.85; p = 0.002), and chronic obstructive pulmonary disease exacerbation (OR, 0.51; 95% CI, 0.29–0.89; p = 0.017) were independently associated with normoxemia.

**Conclusion:**

Monitoring potentially critically ill patients receiving oxygen therapy in wards is essential to mitigate hyperoxemia and optimize the use of healthcare resources. Further research could focus on developing strategies to accomplish this objective.

## 1. Introduction

Oxygen supplementation is a common therapeutic intervention for critically ill patients [1,2], as well as for patients in the perioperative period [3]. However, inappropriate use of supplemental oxygen has been shown to induce hyperoxemia, a condition associated with adverse outcomes and inefficient use of healthcare resources [1,4,5].

The pathophysiological mechanism of oxygen toxicity involves an increased production of reactive oxygen intermediates [6], which cause cellular damage or death by reacting with essential intracellular molecules and overwhelming antioxidant defenses [6,7]. An autopsy series of adult patients with chronic obstructive pulmonary disease (COPD) who had received long-term oxygen therapy revealed pathological findings, including interstitial fibrosis, capillary proliferation, and epithelial hyperplasia in the lungs [8]. In healthy volunteers, the first symptoms of oxygen toxicity can manifest within a few hours of exposure to high concentrations of oxygen [9]. The physiological effects of supplemental oxygen at high concentrations are characterized by an increase in the ratio of dead space to tidal volume, an increase in the right-to-left shunt fraction, and a decrease in diffusing capacity and lung compliance [10–12]. In addition, bleomycin has been shown to exacerbate the development of diffuse alveolar damage in patients with hyperoxemia [13]. Accentuation of hypercapnia is another effect of supplemental oxygen therapy in COPD [14]. In the cardiovascular system, hyperoxemia is associated with decreased cardiac output, increased vascular resistance, and coronary vasoconstriction, particularly in patients with heart failure [15,16]. Hyperoxemia may also be associated with an increased risk of generalized tonic-clonic seizures [17]. In the pediatric population, direct toxicity of supplemental oxygen has been associated with retinopathy of prematurity [18] and bronchopulmonary dysplasia [19]. A previous study, which included nonsurgical patients, revealed that a higher risk of hyperoxemia was independently associated with age and the presence of chronic kidney disease. Conversely, a higher sequential organ failure assessment (SOFA) score, late-night admission (from midnight to 07:00 AM), and primary reasons for admission—including renal/metabolic, neurologic, digestive, and soft tissue/skin/orthopedic conditions—were found to be independently associated with low rates of hyperoxemia [20]. However, despite the well-documented effect of hyperoxemia on outcomes, particularly in patients in the intensive care units (ICUs) [4,21,22] or undergoing surgery [23–26], there is a lack of data on potentially critically ill patients with hyperoxemia who are treated on the ward.

Therefore, we designed the present study to determine the incidence of hyperoxemia and its effect on patient outcomes in potentially critically ill patients treated in hospital wards, and to elucidate the factors associated with hyperoxemia in this population.

## 2. Materials and methods

### 2.1. Study design, setting, and selection of participants

This retrospective cohort study was conducted at Dokuz Eylül University Hospital following approval from the local ethics committee (date: 30/11/2022; IRB number: 2022/38-08). The study population comprised potentially critically ill adult patients (aged 18 years or older) who underwent evaluation by an intensivist to ascertain the necessity of treatment in critical care settings while receiving treatment on hospital wards between 1 January 2020 and 31 December 2020. The patients were grouped as follows: hypoxemic, normoxemic, and hyperoxemic, according to the level of partial pressure of oxygen (PaO_2_) in arterial blood gas analysis performed on the day of intensivist consultation. Hypoxemia was defined as a PaO_2_ < 60 mmHg, normoxemia as 60 mmHg ≤ PaO_2_ < 120 mmHg, and hyperoxemia as PaO_2_ ≥ 120 mmHg. Patients with hypoxemia, a diagnosis of Coronavirus disease 2019 (COVID-19) or post-COVID-19 conditions, a hospital stay shorter than 1 day, missing data on respiratory support, or those who were inadvertently consulted were excluded from the study. The study was conducted in accordance with the ethical standards outlined in the Declaration of Helsinki [27]. The written informed consent was waived due to the retrospective nature of the study.

### 2.2. Variables

The data were collected from each patient’s paper charts and electronic medical records. The patient characteristics—including demographic data, comorbidities, chronic respiratory failure requiring respiratory support, and prognostic scores such as the Glasgow coma scale, Charlson comorbidity index (CCI), and SOFA scores—documented on the day of consultation were recorded. The dataset also encompassed the presence, type, and underlying cause of acute respiratory failure; the type of respiratory support provided in the ward on the day of consultation, along with the level of fraction of inspired oxygen (FiO_2_); and the patients’ laboratory data. Furthermore, hospitalization characteristics—including the ward where the patients stayed, the length of hospital stay, and the need for transfer to the ICU—were recorded. Additionally, the rates of in-hospital mortality, as well as the mortality rates at 28 days, 90 days, 6 months, and 1 year, were documented.

### 2.3. Outcomes

The primary outcome of this study was to determine the incidence of hyperoxemia in patients deemed potentially critically ill and receiving treatment in hospital wards. The secondary outcomes were to identify the independent factors associated with hyperoxemia and to define the impact of hyperoxemia on the length of hospital stay, the need for ICU admission, in-hospital mortality, and mortality at early and long-term follow-up in the study population.

### 2.4. Statistical analysis

The statistical analysis was conducted using the SPSS statistics software (version 29.0.1.0; IBM Corp., Armonk, NY, USA). Categorical variables were presented as numbers and percentages. Continuous variables were presented as the median and interquartile range. To compare continuous and categorical variables in the univariate analysis, the Mann–Whitney U test and chi-square or Fisher’s exact tests were employed, respectively. Binary logistic regression analysis was conducted to ascertain the independent factors associated with hyperoxemia in potentially critically ill patients who were receiving treatment in hospital wards. A model was constructed using the enter method in stepwise logistic regression analysis. This model was specifically designed to incorporate variables linked to hyperoxemia and could impact the results. For each independent factor, the analysis yielded an adjusted odds ratio (OR) and a 95% confidence interval (CI). A two-tailed p-value of less than 0.05 was considered as statistically significant.

## 3. Results

### 3.1. Patient and hospitalization characteristics

Initially, a total of 3846 consultations were screened, and following the exclusion of repetitions, 2148 patients were subjected to analysis (Figure). Of the remaining patients, 396 were excluded due to a diagnosis with or postinfection complications associated with COVID-19. An additional 191 patients were excluded due to the presence of hypoxemia (PaO_2_ < 60 mmHg), while 368 patients were excluded due to a lack of clinical or laboratory data, a hospital stay shorter than 1 day, and other reasons. The final cohort consisted of 1193 patients. Among the patients included in the study, 503 (42.2%) exhibited hyperoxemia, as determined by arterial blood gas analysis performed on the day of consultation.

The patient and hospitalization characteristics are presented in Table 1. The median age of the patients was 73 (63–83) years, and 54.1% of them were male. The median values of the CCI and the SOFA score were 6 (4–8) and 6 (4–9), respectively. Univariate analysis revealed that the proportion of patients with a CCI greater than 6 was significantly higher in the normoxemia group compared to the hyperoxemia group. Most of the patients had at least one comorbid condition with the most frequent comorbidities being hypertension (53.7%), malignancy (35.6%), and diabetes mellitus (30.8%). The proportion of the patients with COPD was significantly higher among those in the normoxemia group than in the hyperoxemia group. The proportion of patients requiring respiratory support at home was notably low. However, the proportion of patients using an oxygen concentrator at home was significantly higher in the normoxemia group than in the hyperoxemia group. The proportion of patients with hyperoxemia was significantly higher among patients treated in the surgical wards, particularly in the orthopedics and neurosurgery wards. Conversely, a significant proportion of patients treated in the emergency department had normoxemia. Furthermore, a notably high rate of hyperoxemia was observed among patients treated in cardiology or neurology wards. Conversely, patients treated in the chest disease ward exhibited a tendency towards normoxemia.

### 3.2. Respiratory characteristics

In Table 2, respiratory characteristics of the patients are presented. The majority of patients (86.1%) exhibited respiratory failure. The most frequent respiratory failure type was hypoxemic (type I), followed by shock-induced (type IV), hypercapnic (type II), and perioperative (type III) respiratory failure. The most frequent causes of respiratory failure were pneumonia (29.0%), pleural effusion (28.8%), and atelectasis (15.2%). Arterial blood gas analysis revealed a higher prevalence of normoxemia compared to hyperoxemia in patients with pneumonia, pleural effusion, and COPD exacerbation. Most patients (56.0%) required positive pressure ventilation, including invasive and noninvasive mechanical ventilation, with rates of 38.9% and 17.1%, respectively. The proportion of patients who received conventional oxygen therapy was 33.6%, while the proportion of those breathing room air was 10.4%. A statistically significant disparity was observed between the normoxemia and hyperoxemia groups in terms of the proportion of patients breathing room air or receiving conventional oxygen therapy. Conversely, the hyperoxemia group exhibited a significantly higher proportion of patients who received positive pressure ventilation or were treated with an FiO_2_ of 50% or more compared to the normoxemia group.

### 3.3. Laboratory data

The laboratory data of patients are presented in Table 3. No statistically significant differences were observed between the normoxemia and hyperoxemia groups regarding the count of inflammatory cells, level of acute phase reactants, liver or kidney function tests, and cardiac enzymes. However, patients with a hemoglobin (Hb) level of ≤ 10.3 g/dL or a partial pressure of carbon dioxide (PCO_2_) of < 30 mmHg exhibited a significantly higher frequency of hyperoxemia than those with higher levels.

### 3.4. Outcomes

As demonstrated in Table 4, the duration of hospitalization was significantly longer in patients with hyperoxemia compared to those with normoxemia (13 [5–25] vs. 9 [3–20] days, p < 0.001). Furthermore, a higher proportion of patients with hyperoxemia were admitted to the ICU compared to those with normoxemia (265 [52.7%] vs. 269 [39.0%], p < 0.001). However, the analysis revealed no differences in in-hospital mortality or mortality rates within the first year between the two groups.

### 3.5. Independent factors associated with hyperoxemia

Table 5 presents the independent factors associated with hyperoxemia. A PCO_2_ level of less than 30 mmHg (OR, 1.61; 95% CI, 1.16–2.25; p = 0.005) in the arterial blood gas analysis; an Hb level of 10.3 g/dL or less (OR, 1.33; 95% CI, 1.01–1.75; p = 0.044); receiving positive pressure ventilation (OR, 1.73; 95% CI, 1.09–2.74; p = 0.021); an FiO_2_ level of 50% or greater (OR, 1.71; 95% CI, 1.10–2.65; p = 0.018); type IV respiratory failure (OR, 1.62; 95% CI, 1.05–2.51; p = 0.030); and receiving treatment in surgical wards (OR, 1.47; 95% CI, 1.02–2.12; p = 0.038) were found to be independently associated with an increased likelihood of hyperoxemia among patients who were potentially critically ill and were treated in wards. Conversely, a CCI score greater than 6 (OR, 0.64; 95% CI, 0.49–0.85; p = 0.002) and COPD exacerbation as an underlying cause of respiratory failure (OR, 0.51; 95% CI, 0.29–0.89; p = 0.017) were found to be independently associated with a tendency towards normoxemia.

## 4. Discussion

The present study examined the risk factors for hyperoxemia in patients deemed potentially critically ill and receiving treatment on hospital wards. The study revealed that the incidence of hyperoxemia was 42.2%, and patients with hyperoxemia exhibited a longer hospital stay and an increased likelihood of requiring ICU admission compared to patients with normoxemia. However, a lack of statistically significant variation was observed in either the early or long-term mortality between patients with normoxemia and hyperoxemia. The study identified several independent risk factors for hyperoxemia, including a PCO_2_ level less than 30 mmHg, a Hb level of 10.3 g/dL or less, positive pressure ventilation, an FiO_2_ level of 50% or greater, type IV respiratory failure, and hospitalization in surgical wards. Conversely, patients with a CCI greater than 6 and COPD exacerbation as a cause of respiratory failure exhibited an independent tendency towards normoxemia.

The present study did not reveal any age-related differences in oxygenation levels among various age groups, either in adults or among elderly patients. However, a study analyzing nonsurgical patients admitted to the hospital revealed that age is an independent factor associated with hyperoxemia [20]. Nevertheless, the alveolar-arterial oxygen gradient rises with age due to a deterioration in ventilation-perfusion mismatch [28]. Additionally, the rise in physiological dead space associated with aging may also contribute to this phenomenon [28]. In this context, the presence of comorbidities, including chronic pulmonary diseases related to airways or parenchymal pathologies and conditions worsening pulmonary perfusion, may play a role in oxygenation. The propensity of patients with COPD and higher scores of CCI to exhibit normoxemia might be explained by these mechanisms. In addition, the predominant tendency among caregivers to administer lower oxygen supplementation to patients with COPD [29] may have also contributed to this outcome.

The ward where the patients were treated also had an effect on oxygenation levels. A higher proportion of patients treated in the emergency department in the normoxemia group may be associated with severe illnesses requiring high-concentration oxygen supplementation. Enhanced monitoring capabilities and frequent use of laboratory tests in the emergency department may help limit the inappropriate use of oxygen. However, the extant literature on oxygen use in adult patients recommends ensuring the availability of pulse oximeters in all locations where emergency oxygen is administered [30]. The necessity for oxygen therapy during the perioperative period is often related to atelectasis, pain, and increased oxygen consumption as a result of shivering [31–34]. The application of multimodal analgesia, positive pressure ventilation, and warming might serve as preventive strategies for requiring high-concentration oxygen supplementation [30,35]. In surgical patients, the target saturation level should be set at 94%–98%, and at 88%–92% for those at risk of hypercapnic respiratory failure [30]. Consequently, clinicians prioritize changing longstanding cultural practices related to supplemental oxygen therapy to prevent hyperoxemia and its adverse effects [5].

The type of respiratory failure had a significant impact on blood oxygen content, with particular attention to the relationship between type IV respiratory failure and hyperoxemia. For patients with type IV respiratory failure, particularly those with acute decompensated heart failure, supplemental oxygen therapy should be administered if peripheral capillary oxygen saturation (SpO_2_) is less than 90%. However, clinicians should avoid routine oxygen therapy in patients without hypoxemia to prevent reduced cardiac output and coronary vasoconstriction [16]. Another underlying cause of type IV respiratory failure, septic shock, has been shown to increase tissue metabolic demand, which needs to be counterbalanced by an increase in tissue oxygen delivery [36]. Inadequate compensation of tissue metabolic demand is a consequence of hemodynamic disturbances that exceed the capacity of cardiovascular compensation, resulting in impaired microcirculation [36,37]. Consequently, the use of oxygen therapy in high concentrations and/or flow rates may become predominant among clinicians treating patients with septic shock or those with worsening clinical status due to impaired metabolic balance related to altered microcirculation and increased tissue oxygen demand. Clinicians should prioritize treatment modalities that provide hemodynamic stability and oxygen delivery sufficient to meet metabolic demand. Until these treatment goals are achieved, patients requiring positive pressure ventilation—particularly those receiving invasive mechanical ventilation with or without an FiO_2_ of 0.50 or greater—should be closely monitored to prevent hyperoxemia in type IV respiratory failure.

The role of the Hb level in oxygen delivery is well-established. The arterial blood oxygen content (CaO_2_) has been demonstrated to exhibit direct proportionality to the Hb, SpO_2_, and PaO_2_ levels (CaO_2_ = [Hb × SpO_2_ × 1.34] + [PaO_2_ × 0.003]) [38]. This association is of particular significance in patients with critical illnesses [39]. An Hb value equal to or lower than the median of 10.3 g/dL in the study population was associated with a significant increase in the proportion of patients with hyperoxemia. In patients with anemia, the signs and symptoms associated with early activation of compensatory mechanisms for tissue perfusion and oxygen delivery may lead clinicians to administer oxygen at high concentrations and flow rates. However, it is imperative to acknowledge that oxygen delivery and tissue perfusion are influenced by a multitude of physiological factors that extend beyond Hb level [40]. Additionally, studies comparing liberal versus conservative Hb thresholds have not demonstrated any increase in mortality among critically ill patients [41,42]. The analysis, based on the hypothesis that hyperventilation becomes clinically recognizable at a PCO_2_ threshold of 30 mmHg, revealed that the proportion of patients with hyperoxemia was significantly higher among those with hypocapnea in the present study. In this context, potentially critically ill patients with tachypnea due to nonpulmonary pathologies are at risk of hyperoxemia due to supplemental oxygen therapy. Moreover, the alveolar partial pressure of oxygen is determined by factors including the FiO_2_ level, atmospheric pressure, water vapor pressure, and the partial pressure of alveolar carbon dioxide. A decrease in alveolar carbon dioxide pressure, particularly in patients with tachypnea, has been demonstrated to result in an increase in the partial pressure of oxygen within the alveoli [43]. Consequently, this results in an increase in arterial blood oxygen content. In this population, it is imperative to investigate the underlying causes of tachypnea and potential pulmonary pathologies to determine the necessity of oxygen support.

The relationship between hyperoxemia and patient outcomes remains a subject of debate in literature. The present study demonstrated that patients with hyperoxemia exhibited prolonged hospital stays and a heightened necessity for treatment in critical care units. This phenomenon may be attributable to the role of hyperoxemia reflecting disease severity, rather than its direct effect on adverse outcomes. Additionally, the observed outcomes may be attributable to the propensity of practitioners to administer oxygen therapy at high concentrations and flows to patients with more severe illness. A systematic review comparing liberal versus conservative oxygen therapy in acutely ill adult patients revealed a dose-dependent increase in early- and long-term mortality in patients treated with liberal oxygen therapy, but no significant difference in hospital-acquired pneumonia, disability, or length of hospital stay [4]. A further study revealed a correlation between hyperoxemia and higher mortality rates, along with reduced ventilator-free days, among critically ill patients, examining both severe hyperoxemia and mild hyperoxemia or normoxemia [21]. However, another randomized trial demonstrated that the number of ventilator-free days and 6 month mortality did not differ significantly between patients receiving liberal or conservative oxygen therapy [44]. A further study demonstrated that hyperoxemia increases the risk of in-hospital mortality by 1.8-fold among patients admitted to critical care following cardiopulmonary resuscitation [22]. However, the present study did not reveal any differences between the groups based on in-hospital, early- or long-term mortality. From the perspective of anesthesiology practices, particularly in the context of cardiac anesthesia, the extant literature suggests a correlation between hyperoxemia and perioperative pulmonary complications [23]. However, this correlation does not extend to the worsening of postoperative cognition [45]. Nevertheless, the impact of hyperoxemia on postoperative kidney function remains a subject of debate [26,46]. Moreover, the earlier metaanalyses indicated that hyperoxemia may have a mitigating effect on the development of surgical site infections among surgical patients [47,48]. However, subsequent randomized controlled trials have failed to corroborate these conclusions [24,25]. It is important to note that these studies have utilized varying definitions of hyperoxemia, which may have contributed to the inconsistent outcomes.

This study has some limitations. The study population comprised patients who were defined as “potentially critically ill patients” and underwent evaluation by an intensivist to determine whether they require treatment in a critical care setting while receiving care in hospital wards. The study exclusively included patients who underwent evaluation by an intensivist to determine the need for treatment in critical care settings after a request by the patients’ attending physician. It is imperative to acknowledge that the study’s scope did not encompass all patients who remained in the hospital wards. Consequently, the study’s findings do not provide unequivocal evidence regarding the incidence of hyperoxemia and its associated risk factors among all patients hospitalized in wards. Additionally, patients were grouped according to PaO_2_ levels on the day they underwent an intensivist evaluation. Further studies encompassing all patients treated in hospital wards are required to generalize the study results. Additionally, the inclusion of patients evaluated in the emergency department may have introduced heterogeneity within the study population based on ventilatory support, monitoring facilities, or severity of illness. Nevertheless, this study has several notable strengths. The investigation of the impact of hyperoxemia on length of hospital stay, need for ICU admission, and early and long-term mortality in this population represents a significant contribution to the field. Furthermore, the study identifies subsets of hyperoxemia-related factors that will contribute to elucidating which patients receiving oxygen and/or ventilatory support should be monitored closely by healthcare practitioners for the potential emergence of hyperoxemia.

In conclusion, hyperoxemia is a condition that clinicians should avoid, not only in patients treated in critical care units or operating rooms, but also in patients receiving treatment in hospital wards with diseases that have the potential to progress to acute critical illness. The optimization of FiO_2_ and/or oxygen flow according to target values of SpO_2_ or PaO_2_ in patients receiving supplemental oxygen therapy has the potential to contribute to the prevention of hyperoxemia and its adverse effects, as well as to the limitation of healthcare costs. Future studies may focus on novel strategies to reduce the incidence of hyperoxemia in patients receiving supplemental oxygen therapy in all settings.

## Figures and Tables

**Figure:**
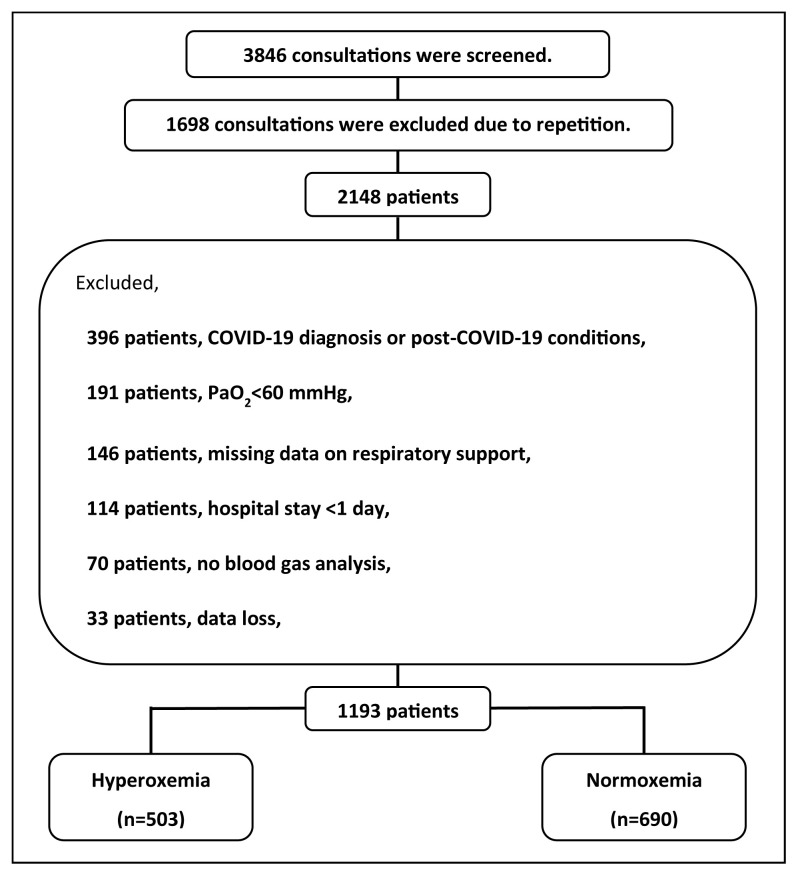
Study flowchart.

**Table 1:** Characteristics of patients and hospitalizations.

Characteristics	All (n = 1193)	Hyperoxemia (n = 503)	Normoxemia (n = 690)	p value
Age	73 (63–83)	73 (62–83)	73 (63–82)	0.81
Adult (18–64 years)	337 (28.2)	145 (28.8)	192 (27.8)	0.75
Younger elderly (65–74 years)	297 (24.9)	125 (24.9)	172 (24.9)	1.00
Older elderly (75–84 years)	321 (26.9)	128 (25.4)	193 (28.0)	0.36
Oldest elderly (≥85 years)	238 (19.9)	105 (20.9)	133 (19.3)	0.51
Sex, men	645 (54.1)	261 (51.9)	284 (55.7)	0.22
CCI	6 (4–8)	6 (4–7)	6 (4–8)	**0.027**
CCI > 6	475 (39.8)	179 (35.6)	296 (42.9)	**0.012**
SOFA score*	6 (4–9)	6 (4–9)	6 (4–9)	0.16
Comorbidity	1100 (92.2)	461 (91.7)	639 (92.6)	0.59
Hypertension	641 (53.7)	278 (55.3)	363 (52.6)	0.38
Malignancy	425 (35.6)	169 (33.6)	256 (37.1)	0.22
Solid organ malignancy	365 (30.6)	144 (28.6)	221 (32.0)	0.23
Hematological malignancy	66 (5.5)	27 (5.4)	39 (5.7)	0.90
Diabetes mellitus	368 (30.8)	142 (28.2)	226 (32.8)	0.10
Coroner artery disease	269 (22.5)	120 (23.9)	149 (21.6)	0.36
Congestive heart failure	202 (16.9)	89 (17.7)	113 (16.4)	0.58
COPD	169 (14.2)	54 (10.7)	115 (16.7)	**0.004**
Chronic kidney disease	164 (13.7)	71 (14.1)	93 (13.5)	0.80
Cerebrovascular disease	151 (12.7)	68 (13.5)	83 (12.0)	0.48
Alzheimer’s disease	136 (11.4)	53 (10.5)	83 (12.0)	0.46
Chronic liver disease	73 (6.1)	25 (5.0)	48 (7.0)	0.18
History of thromboembolism	32 (2.7)	10 (2.0)	22 (3.2)	0.28
Obesity	16 (1.3)	6 (1.2)	10 (1.4)	0.80
Respiratory support at home				
Home-BIPAP/CPAP	30 (2.5)	8 (1.6)	22 (3.2)	0.09
Oxygen concentrator	70 (5.9)	20 (4.0)	50 (7.2)	**0.018**
Nebulizer	76 (6.4)	29 (5.8)	47 (6.8)	0.55
Ward where the patients stayed				
Emergency department	551 (46.2)	205 (40.8)	346 (50.1)	**0.001**
Medical wards	414 (34.7)	181 (36.0)	233 (33.8)	0.46
Internal medicine±	253 (21.2)	100 (19.9)	153 (22.2)	0.35
Chest disease	86 (7.2)	27 (5.4)	59 (8.6)	**0.041**
Cardiology	30 (2.5)	23 (4.6)	7 (1.0)	**<0.001**
Neurology	27 (2.3)	21 (4.2)	6 (0.9)	**<0.001**
Infectious disease	14 (1.2)	8 (1.6)	6 (0.9)	0.28
Dermatology	4 (0.3)	2 (0.4)	2 (0.3)	1.00
Surgical wards	228 (19.1)	117 (23.3)	111 (16.1)	**0.002**
General surgery	102 (8.5)	45 (8.9)	57 (8.3)	0.68
Orthopedics	54 (4.5)	33 (6.6)	21 (3.0)	**0.005**
Urology	22 (1.8)	12 (2.4)	10 (1.4)	0.29
Neurosurgery	31 (2.6)	19 (3.8)	12 (1.7)	**0.041**
Cardiovascular surgery	12 (1.0)	5 (1.0)	7 (1.0)	1.00
Reconstructive surgery	4 (0.3)	0 (0.0)	4 (0.6)	0.14
Ear, nose, and throat surgery	3 (0.3)	3 (0.6)	0 (0.0)	0.08

All values are expressed as median and interquartile range, or as number and percentage.

Abbreviations: CCI, Charlson comorbidity index; SOFA score, sequential organ failure assessment score; COPD, chronic obstructive pulmonary disease; BIPAP, bilevel positive airway pressure; CPAP, continuous positive airway pressure.

* Calculated based on data collected on the day of consultation.

± Includes medical oncology, hematology, gastroenterology, nephrology, general internal medicine, and geriatrics wards.

**Table 2:** Respiratory pathologies and support characteristics.

Characteristics	All (n = 1193)	Hyperoxemia (n = 503)	Normoxemia (n = 690)	p value
Presence of respiratory failure	1027 (86.1)	446 (88.7)	581 (84.2)	**0.028**
Type I	532 (44.6)	202 (40.2)	330 (47.8)	**0.008**
Type II	164 (13.7)	56 (11.1)	108 (15.7)	**0.030**
Type III	120 (10.1)	61 (12.1)	59 (8.6)	**0.038**
Type IV	165 (13.8)	92 (18.3)	73 (10.6)	**<0.001**
Causes of respiratory failure*				
Pneumonia	298 (29.0)	90 (20.2)	208 (35.8)	**<0.001**
Pleural effusion	296 (28.8)	108 (24.2)	188 (32.4)	**0.004**
Atelectasis	156 (15.2)	64 (14.3)	92 (15.8)	0.54
Pulmonary edema	112 (10.9)	43 (9.6)	69 (11.9)	0.29
COPD exacerbation	82 (8.0)	22 (4.9)	60 (10.3)	**0.002**
Pulmonary aspiration	57 (5.6)	19 (4.3)	38 (6.5)	0.13
Pulmonary embolism	51 (5.0)	24 (5.4)	27 (4.6)	0.66
Pneumothorax	31 (3.0)	16 (3.6)	15 (2.6)	0.36
Interstitial lung disease	20 (1.9)	5 (1.1)	15 (2.6)	0.11
ARDS	19 (1.9)	6 (1.3)	13 (2.2)	0.36
Pulmonary contusion	19 (1.9)	9 (2.0)	10 (1.7)	0.82
Bronchiectasis	7 (0.7)	1 (0.2)	6 (1.0)	0.15
Hemothorax	7 (0.7)	5 (1.1)	2 (0.3)	0.25
Alveolar hemorrhage	5 (0.5)	2 (0.4)	3 (0.5)	1.00
Sarcoidosis	2 (0.2)	0 (0.0)	2 (0.3)	0.51
Inhalation injury	1 (0.1)	1 (0.2)	0 (0.0)	0.43
Type of respiratory support				
Breathing room air	124 (10.4)	17 (3.4)	107 (15.5)	**<0.001**
Conventional oxygen therapy	401 (33.6)	139 (27.6)	262 (38.0)	**<0.001**
Nasal cannula	163 (13.7)	46 (9.1)	117 (17.0)	**<0.001**
Oxygen mask	226 (18.9)	90 (17.9)	136 (19.7)	0.46
Mask with reservoir	12 (1.0)	3 (0.6)	9 (1.3)	0.26
Positive pressure ventilation	668 (56.0)	347 (69.0)	321 (46.5)	**<0.001**
IMV	464 (38.9)	268 (53.3)	196 (28.4)	**<0.001**
NIMV	204 (17.1)	79 (15.7)	125 (18.1)	0.31
FiO_2_, %	50.0 (35.0–50.0)	50.0 (40.0–50.0)	40.0 (35.0–50.0)	**<0.001**
FiO_2_ ≥ 50%	599 (50.2)	318 (63.2)	281 (40.7)	**<0.001**

All values are expressed as median and interquartile range, or as number and percentage.

Abbreviations: COPD, chronic obstructive pulmonary disease; ARDS, acute respiratory distress syndrome; IMV, invasive mechanical ventilation; NIMV, noninvasive mechanical ventilation; FiO_2_, fraction of inspired oxygen.

* The distribution of respiratory failure etiologies is presented exclusively based on the number of patients with respiratory failure, rather than the total number of patients.

**Table 3:** Laboratory data of the patients.

Laboratory tests*	All (n = 1193)	Hyperoxemia (n = 503)	Normoxemia (n = 690)	pvalue
WBC, × 10^3^/μL	11.9 (8.0–16.6)	11.6 (7.9–15.8)	12.0 (8.1–17.2)	0.18
Neutrophil, × 10^3^/μL	9.5 (6.0–14.0)	9.5 (5.7–13.2)	9.5 (6.1–14.4)	0.36
Hemoglobin, g/dL	10.3 (8.8–12.0)	10.1 (8.7–11.7)	10.6 (9.0–12.4)	**0.002**
Hemoglobin ≤ 10.3 g/dL	611 (51.2)	281 (55.9)	330 (47.8)	**0.007**
Lymphocyte, × 10^3^/μL	0.9 (0.5–1.5)	0.9 (0.5–1.4)	0.9 (0.5–1.5)	0.59
Platelet, × 10^3^/μL	204.0 (125.0–293.0)	209.0 (126.0–300.0)	201.0 (122.0–289.0)	0.39
HS Troponin I, ng/L	35.3 (13.1–153.8)	34.0 (13.8–162.1)	36.5 (12.6–142.0)	0.59
D-dimer, ug/mL	4.5 (1.9–11.0)	5.1 (2.2–11.5)	4.2 (1.7–10.3)	0.14
BNP, pg/mL	344.0 (104.3–844.1)	391.8 (116.5–916.4)	296.0 (99.8–833.0)	0.17
BUN, mg/dL	31.5 (19.7–56.7)	32.2 (18.7–55.5)	31.1 (20.0–56.8)	0.86
Creatinine, mg/dL	1.18 (0.71–2.23)	1.24 (0.74–2.3)	1.16 (0.70–2.15)	0.37
Total Bilirubin, mg/dL	0.92 (0.62–1.50)	0.90 (0.61–1.51)	0.92 (0.63–1.48)	0.40
AST, U/L	37 (23–81)	40 (23–83)	37 (24–80)	0.84
ALT, U/L	24 (14–52)	24 (14–51)	24 (14–53)	0.83
LDH, U/L	317 (231–499)	312 (227–488)	320 (232–516)	0.53
ALP, U/L	94 (67–151)	96 (67–149)	94 (68–152)	0.93
CRP, mg/L	105 (33–187)	102 (37–179)	110 (31–192)	0.40
Procalcitonin, ng/mL	0.39 (0.17–2.57)	0.38 (0.16–2.41)	0.39 (0.18–2.64)	0.64
Blood gas analysis				
pH	7.38 (7.27–7.45)	7.37 (7.28–7.45)	7.38 (7.27–7.44)	0.52
PCO_2_, mmHg	36 (30–45)	35.3 (28.0–43.4)	37.0 (30.5–46.0)	**0.005**
PCO_2_ < 30 mmHg	284 (23.8)	135 (26.8)	149 (21.6)	**0.039**
PaO_2_, mmHg	111 (83–146)	152.0 (134.0–189.0)	88.5 (73.5–105.0)	**< 0.001**
HCO_3_, mEq/L	22.0 (17.8–26.2)	21.8 (17.9–26.0)	22.4 (17.7–26.4)	0.26
Base excess	−1.8 (−7.2–2.3)	−2.5 (−7.6–2.0)	−1.5 (−7.0–2.4)	0.16
Lactate, mmol/L	1.8 (1.2–3.6)	1.9 (1.2–3.9)	1.8 (1.2–3.4)	0.31
SO_2_, %	98.0 (95.8–99.0)	98.9 (98.1–99.1)	96.5 (94.0–98.0)	**< 0.001**
PaO_2_/FiO_2_	276.0 (207.5–368.0)	346.0 (290.0–425.0)	220.0 (172.0–291.0)	**< 0.001**

All values are expressed as median and interquartile range.

Abbreviations: WBC, white blood cell; hS-Troponin I, high-sensitivity troponin I; BNP, brain natriuretic peptide; BUN, blood urea nitrogen; AST, aspartate aminotransferase; ALT, alanine aminotransferase; LDH, lactate dehydrogenase; ALP, alkaline phosphatase; CRP, C-reactive protein; PCO_2_, partial pressure of carbon dioxide; PaO_2_, partial pressure of oxygen; SO_2_, oxygen saturation; FiO_2_, fraction of inspired oxygen.

* Measured on the day of consultation.

**Table 4:** Outcomes.

Characteristics	All (n = 1193)	Hyperoxemia (n = 503)	Normoxemia (n = 690)	pvalue
Length of hospital stay, days	11 (4–22)	13 (5–25)	9 (3–20)	**< 0.001**
ICU admission	534 (44.8)	265 (52.7)	269 (39.0)	**< 0.001**
In-hospital mortality	555 (46.5)	246 (48.9)	309 (44.8)	0.18
28-day mortality	520 (43.6)	215 (42.7)	305 (44.2)	0.64
90-day mortality	659 (55.2)	285 (56.7)	374 (54.2)	0.41
6-month mortality	695 (58.3)	301 (59.8)	394 (57.1)	0.37
1-year mortality	714 (59.8)	306 (60.8)	408 (59.1)	0.59

All values are expressed as median and interquartile range, or as number and percentage.

Abbreviations: ICU, intensive care unit.

**Table 5:** Independent factors associated with hyperoxemia.

Factors related to hyperoxemia	OR (95% CI)	p*v*alue
**Comorbidity**		
CCI > 6	0.64 (0.49–0.85)	**0.002**
**Laboratory data**		
PCO_2_ < 30 mmHg	1.61 (1.16–2.25)	**0.005**
Hb ≤ 10.3 g/dL	1.33 (1.01–1.75)	**0.044**
**Underlying reason for respiratory failure**		
COPD exacerbation	0.51 (0.29–0.89)	**0.017**
**Respiratory support**		
Positive pressure ventilation	1.73 (1.09–2.74)	**0.021**
FiO_2_ ≥ 50%	1.71 (1.10–2.65)	**0.018**
**Type of respiratory failure**		
Type IV	1.62 (1.05–2.51)	**0.030**
Type I	0.93 (0.66–1.31)	0.68
**Wards**		
Surgical wards	1.47 (1.02–2.12)	**0.038**

Abbreviations: CCI, Charlson comorbidity index; PCO_2_, partial pressure of carbon dioxide; Hb, hemoglobin; COPD, chronic obstructive pulmonary disease; FiO_2_, fraction of inspired oxygen.
